# Determinants of Salivary Cotinine among Smokeless Tobacco Users: A Cross-Sectional Survey in Bangladesh

**DOI:** 10.1371/journal.pone.0160211

**Published:** 2016-08-09

**Authors:** Rumana Huque, Sarwat Shah, Nasir Mushtaq, Kamran Siddiqi

**Affiliations:** 1 Department of Economics, University of Dhaka, Dhaka, Bangladesh; 2 Department of Health Sciences, University of York, Heslington, York, United Kingdom; 3 Department of Biostatistics and Epidemiology, University of Oklahoma Health Sciences Center, Tulsa, United States of America; Dartmouth College Geisel School of Medicine, UNITED STATES

## Abstract

**Introduction:**

More than 80% of all smokeless tobacco (ST) products in the world are consumed in South Asia; yet little is known about their consumption behaviour, addictiveness, and toxic properties. This paper, for the first time, describes associations between salivary cotinine concentrations among ST users in Bangladesh and their socio-demographic characteristics and tobacco use behaviours.

**Methods:**

In a survey of ST users in Dhaka, Bangladesh, we purposively recruited 200 adults who were non-smokers but consumed ST on a regular basis. In-person interviews were conducted to obtain information about socio-demographic and ST use behaviours, and saliva samples were collected to measure cotinine concentration. Simple and multiple linear regression analyses were conducted to test associations between the log transformed salivary cotinine concentration and other study variables.

**Results:**

The geometric mean of cotinine concentration among ST users was 380ng/ml (GSD:2). Total duration of daily ST use in months had a statistically significant association with cotinine concentration. Other ST use characteristics including type and quantity of ST use, swallowing of tobacco juice, urges and strength of urges and attempts to cut down on tobacco use were not found to be associated with cotinine concentration in a multivariable model.

**Conclusion:**

This is the first report from Bangladesh studying cotinine concentration among ST users and it points towards high levels of addiction. This warrants effective tobacco control policies to help ST cessation and prevention.

## Introduction

Almost a fifth of world’s tobacco is consumed in smokeless form [1], mostly in South Asia. Smokeless tobacco (ST) use is integral to South Asian culture, being incorporated in their traditional values, spirituality, beliefs, festivals, marriage ceremonies, lifestyle, rituals and popular entertainment [2–4]. Over 250 million people use ST products in South East Asia of which 13% reside in Bangladesh [5]. In Bangladesh, 27.2% (25.9 million) adults, 26.4% (12.5 million) of men and 27.9% (13.4 million) of women, are current consumers of ST products [6]. Thus ST use prevalence exceeds cigarette smoking among both men and women in this population.

ST products are considered highly addictive due to their high nicotine concentration [7]. ST products also contain carcinogenic substances like tobacco-specific N-nitrosamines (TSNAs) leading to an increased risk of cancers of oral cavity, pharynx, and oesophagus. Increased risk of cardiovascular disease mortality has also been observed among ST users [8]. ST consumption during pregnancy is associated with low birth weight and stillbirths [1], [9].

A diverse range of ST products are available worldwide, varying in their composition, methods of preparation and consumption, and associated health risks. Khaini, Zarda and Gutkha are popular in South and South-East Asia. In other parts of world, the most commonly used ST products include Snus (Nordic countries and North America), Chimó (Venezuela), Nass (Uzbekistan, Kyrgyzstan), Tambook (Sudan, Chad), and Snuff (Nigeria, Ghana, South Africa) [10]. Depending on the type of ST product, nicotine content may vary, and therefore, measurement of nicotine and its metabolites among ST users is important to understand the addictive potential of ST products. Cotinine is a sensitive and specific quantitative indicator of the uptake of nicotine over the past few days [11]. Its concentration in body fluids is determined by the rate of nicotine metabolism and cotinine clearance [12], [13]. Although individual variation might exist in salivary cotinine concentrations because of such parameters, it is still an important indicator of nicotine intake and a predictor of nicotine dependence. A few US-based studies have explored association between cotinine concentration among ST users and their tobacco consumption behaviours. These included years of ST use, quantity, total dipping time, swallowing of tobacco juices, previous quit attempts, and demographic factors, including age, marital status, and occupation [12–15]. However, such evidence is lacking among ST users in South Asia–a region with the highest ST disease burden. Due to the distinct nature of ST products consumed in South Asia, it is important to study factors associated with cotinine concentration among ST users in this region. In this study we examined association between salivary cotinine concentration among ST users in Bangladesh and their socio-demographic characteristics and tobacco use behaviours. To our knowledge, this is the first study reporting salivary cotinine concentration among ST users in Bangladesh.

## Methods

### Study design and study participants

A cross-sectional survey was carried out recruiting a purposive sample of ST consumers in Dhaka, Bangladesh.

We recruited 200 current ST users aged 18 years or older, who were non-smokers (either never smokers or former smokers who have not smoked for at least past one year). Current ST users were defined as those using ST products for at least one year, with a minimum of one can or pouch of ST used per week. Those with a history of other substance abuse or psychiatric illnesses were excluded. Participants were recruited purposively from two urban cities in Dhaka, namely Mohakhali and Mirpur, through convenience sampling techniques, including snowball sampling and community referrals. Trained field researchers recruited participants by visiting houses in these communities, at community gatherings, ST shops, and through personal referrals. Researchers first approached local leaders in the study areas and people buying ST products from shops and explained the study objectives. Local leaders and ST users gave references of their relatives, friends, and neighbours who use ST. We recruited male and female ST users in equal proportion. Taka 200 (GBP 1.5) was paid to respondents as opportunity cost of their time. Interviews and saliva sample collections were conducted at ST users’ home or other mutually convenient place, such as their workplace. Informed written consent was obtained from all study participants. Data were collected between September and November 2014. This study was approved by the Bangladesh Medical Research Council (BMRC) and the Research Governance Committee at the University of York, UK.

### Measures

A survey containing a set of questionnaires was administered by trained interviewers to collect information on the; (a) socio-demographic characteristics, including age, sex, education, and household asset ownership; and b) tobacco use behaviours, i.e. quantity (in grams) of ST used per week, number of chews/dips per day (DPD) in the past week, duration of ST use in months, type of ST products used, swallowing of tobacco juices, urge and strength of urge to use ST, Fagerström Test for Nicotine Dependence (FTND- ST), Tobacco Dependence Screener (TDS), attempt to cut down on ST use in the past, and past smoking status. ST products are generally sold in cans and packets that vary in weights. We standardized our quantity estimates by showing participants cans and packets of 10, 20 and 30 grams. We asked the respondent to point out to the size of cans and packets they generally buy and how long does it take for them to consume these. From this information, we estimated the amount of ST in grams consumed during an average week.

Measures of dependence, FTND-ST and TDS were adapted through the process of translation and back-translation, and cross-cultural validation for use in Bangladeshi culture and language. Translation and back-translation was done by bilingual and bicultural translators outside the research team [16].

To measure cotinine concentration, saliva samples were collected from all participants at the interviews using Salivette, oral swab and swab storage tube. Saliva samples were taken at least 30 minutes after eating, drinking or taking medication. A sterile cotton swab was tipped under the tongue without touching, and was left there until it became soggy. This took up to five minutes. The swab was then directly spat into the kit and closed firmly with the stopper. The saliva samples were kept at the study centre at room temperature for a maximum of four days before being shipped to the UK and subsequently tested for cotinine at ABS Laboratories, UK. The samples were frozen at -20 degrees immediately on receipt in the UK and then thawed for analysis. Cotinine was determined using a validated Liquid chromatography-tandem mass spectrometry (LC-MS/MS) assay in human saliva using cotinine-d3 to internally standardize the procedure. The method with a calibration range of 1 to 750 ng/mL was applied to this study as the samples were from subjects that chewed tobacco. Cotinine was extracted from human saliva using 50 μL of sample using liquid/liquid extraction with ethyl acetate. The solvent was removed under nitrogen and the extract re-suspended in methanol for quantitative determination using Hydrophilic interaction liquid chromatography (HILIC) on a Thermo perfluorinated phenyl (PFP) column using LC-MS/MS with multiple reaction monitoring (MRM) on an Agilent 1100 LC system interfaced to an ABSciex API 4000 tandem mass spectrometer. Analytical acceptance criteria were considered as specified by the Food and Drug Administration [14].

### Statistical analysis

Exploratory analysis of all variables was performed. Geometric mean was calculated for salivary cotinine concentration across socio-demographic and tobacco use behaviours. We also explored whether differences in proportion of tobacco use behaviours exist across sex of the participants. Simple and multiple linear regression analyses were conducted to compute crude and adjusted estimates for association of variables with saliva cotinine levels. Assumption of normality was not met for the bivariate analysis and saliva cotinine levels were positively skewed; therefore we applied logarithmic transformation to salivary cotinine levels for statistical analyses. Regression diagnostics were performed to evaluate multicollinearity and influential observations. We set the cut off value of Variance Inflation Factor >5 to exclude the factors from the final model [17]. None of the variables met this criterion. However only those variables that were significantly associated with cotinine levels in simple linear regression were included in the final model due to the limited sample size.

All analyses were performed using SAS v.9.4 (SAS Institute Inc. Cary, NC, USA) and a level of 0.05 was used for statistical significance.

## Results

We screened 224 adult ST users for eligibility; out of which 200 were recruited in the study. The mean age of participants was 46.8 (SD:13.3) and 50% of them were males. The geometric mean salivary cotinine concentration among these ST users was 380ng/ml. All participants used ST every day in the past seven days while mean number of DPD was 12 (SD: 6). Table 1 summarizes tobacco use behaviours by sex.

**Table 1:** Tobacco use behaviours of the study participants (n = 200).

Tobacco use behaviour	Overall *n* (%)	Male (n = 100) *n* (%)	Female (n = 100) *n* (%)	p-value
Duration of daily ST use †	226 (134)	207 (126)	244 (141)	.05
Dips per day †	11.7 (6)	12.8 (6.7)	10.5 (4.4)	.004
Quantity of ST use				.20
< 20	105 (53)	53 (53)	52 (52)	
20–30	74 (37)	33 (33)	41 (41)	
>30	21 (11)	14 (14)	7 (7)	
Time to first chew (TTF)				< .001
> 60 mins	74 (37)	50 (50)	24 (24)	
31–60 mins	40 (20)	26 (26)	14 (14)	
6–30 mins	49 (25)	16 (16)	33 (33)	
< 5 minutes	37 (18)	8 (8)	29 (29)	
FTND-ST†	4.7 (2.5)	3.7 (2.1)	5.7 (2.4)	< .001
TDS based dependence				< .001
Yes	117 (59)	77 (77)	40 (40)	
No	83 (41)	23 (23)	60 (60)	
Swallowing of tobacco juices				.04
Never	14 (7)	9 (9)	5 (5)	
Sometimes	106 (53)	62 (62)	44 (44)	
Always	80 (40)	29 (29)	51 (51)	
Urge to use ST				.01
Not at all to sometime	22 (11)	17 (17)	5 (5)	
A lot of time to all the time	178 (89)	83 (83)	95 (95)	
Strength of urges				.01
Not at all to sometime	13 (7)	11 (11)	2 (2)	
A lot of time to all the time	187 (93)	89 (89)	98 (98)	
Past attempts to decrease ST use				.01
Yes	43 (22)	14 (14)	29 (29)	
No	157 (78)	86 (86)	71 (71)	
Past smoking status				< .001
Smokers	40 (20)	37 (37)	3 (3)	
Never smokers	160 (80)	63 (63)	97 (97)	
Type of ST products used				< .001
Saada patta/Gul/Khaini	15 (8)	9 (9)	6 (6)	
Paan with Zarda	137 (69)	82 (82)	55 (55)	
Combination of two or more	48 (24)	9 (9)	39 (39)	

Note: FTND-ST = Fagerström Test for Nicotine Dependence–Smokeless Tobacco; TDS based dependence = Tobacco Dependence Screener score of 5 or more; Quantity of ST use = Grams of ST used per week

† Statistics reported are mean (*M*) and standard deviation (*SD*)

Tables 2 and 3 present parameter estimates and their 95% CIs from simple linear regression examining associations between ST dependence and participants’ socio-demographic variables and ST use behaviours, respectively. The analyses suggested that among socio-demographic characteristics and tobacco use behaviours; education, DPD, duration of daily ST use in months, types of ST used and TTF and FTND-ST scores were associated with log mean cotinine concentration reaching statistical significance. Variables such as swallowing of tobacco juices and smoking status were not associated with cotinine concentration.

**Table 2:** Association between socio-demographic characteristics and salivary cotinine concentration^*^.

Variable	n (%)	Cotinine ^†^ (ng/ml)	β-Coefficient (95%CI)	Standard Error	p–value
Sex					
Female	100 (50)	361	Ref	.11	.38
Male	100(50)	399	.09 (-.12 to .30)		
Education					
No formal education	95 (47)	426	Ref		
Primary or less	75 (37)	270	.06 (-.29 to .16)	.11	.58
Secondary or less	20 (10)	382	-.44 (-.80 to -.07)	.18	.02
High secondary or more	12 (6)	401	-.06 (-.43 to .54)	.24	.82
SES index			.001 (-.004 to .01)	.002	.61

* Log transformation of salivary cotinine concentration

† Geographic mean

**Table 3:** Overall association between ST use behaviors and salivary cotinine concentration^*^ (simple linear regression).

Tobacco use behaviour	*n (%)*	Cotinine ^†^ (ng/ml)	β-Coefficient (95%CI)	Standard Error	p–value
Duration of daily ST use			.001 (.0001 to .002)	.00	.02
Dips per day			.02 (.01 to .04)	.01	.02
Quantity of ST use					
< 20	105 (52)	340	Ref	-	-
20–30	74 (37)	417	.20 (-.03 to .42)	.11	.08
> 30	21 (11)	473	.33 (-.02 to (.68)	.17	.06
Time to first chew (TTF)					
> 60 mins	74 (37)	315	Ref	-	-
31–60 mins	40 (20)	380	.20 (-.09 to .48)	.15	.18
6–30 mins	48 (24)	434	.31 (.05 to .58)	.14	.02
< 5 minutes	37 (19)	461	.38 (.10 to .68)	.14	.01
FTND-ST†			.7	.02	.11
TDS based dependence					
Yes	117 (59)	375	Ref	-	-
No	83 (41)	386	.04 (-.17 to .25)	.11	.69
Swallowing of tobacco juices					
Never	14 (7)	357	Ref	-	-
Sometimes	106 (53)	355	-.01 (-.42 to .41)	.21	.98
Always	80 (40)	419	.15 (-.27 to .58)	.21	.48
Urge to use ST					
Not at all to sometime	22 (11)	340	Ref	-	-
A lot of time to all the time	178 (81)	385	.12 (-.21 to .45)	.17	.48
Strength of urges					
Not at all to sometime	13 (7)	374	Ref	-	-
A lot of time to all the time	187 (93)	380	.01 (-.41 to .43)	.21	.96
Past attempts to decrease ST use					
Yes	43 (21)	318	Ref	-	-
No	157 (79)	398	.22 (-.03 to .47)	.13	.08
Past smoking status					
Smokers	40 (20)	390	Ref	-	-
Never smokers	160 (80)	377	-.03 (-.30 to .22)	.13	.76
Type of ST products used					
Saada patta/Gul/Khaini	15 (7)	338	Ref	-	-
Paan with Zarda	137 (68)	349	.23 (-.17 to .62)	.20	.26
Combination of two or more	50 (25)	460	.26 (.02 to .51)	.12	.04

Note: FTND-ST = Fagerström Test for Nicotine Dependence–Smokeless Tobacco; TDS based dependence = Tobacco Dependence Screener score of 5 or more; Quantity of ST use = Grams of ST used per week

* Log transformation of salivary cotinine concentration

† Geographic mean

Multiple linear regression model, presented in Table 4, suggested that DPD was the only significant variable while adjusting for duration of daily ST use in months, types of ST products, TTF, and FTND-ST.

**Table 4:** Adjusted association between ST use behaviours and salivary cotinine concentration^*^ (multiple linear regression).

Tobacco use behaviour	β-Coefficient (95%CI)	Standard Error	p–value
Duration of daily ST use	.001	.00	.15
Dips per day	.02	.01	.03
Time to first chew (TTF)			
> 60 mins	Ref		
31–60 mins	.10	.15	.49
6–30 mins	.21	.19	.28
< 5 minutes	.16	.24	.51
FTND-ST†	.01	.00	.79
Type of ST products used			
Saada patta/Gul/Khaini	Ref		
Paan with Zarda	.07	.21	.8
Combination of two or more	.11	.14	.5

* Log transformation of salivary cotinine concentration

† Geographic mean

Results suggested that salivary cotinine concentration increased by 0.2 ng/ml (p = 0.04) with each additional dip of ST per day while holding the effect of other factors constant.

## Discussion

This is the first study to describe associations between salivary cotinine concentrations and socio-demographic characteristics and tobacco use behaviours among ST users in Bangladesh. We found that ST users in our study have very high levels of cotinine (380ng/ml [GSD:2]) compared to cigarette smokers as found in a previous study [18].

Previous studies of ST users have identified distinct tobacco use characteristics associated with cotinine concentration. Some tobacco use characteristics such as cans of tobacco consumed per week, swallowing of tobacco juice, and past quit attempts were reported to be associated with cotinine in previous studies [12], [13] but this study could not find such associations. This study evaluated univariate and multivariate associations between cotinine concentration and tobacco use characteristics. We found duration of daily ST use in months as the only factor to have an independent significant association with salivary cotinine. Similar association was found in a past study of regular ST users in the US [19]. The differences in findings of this study may be attributable to the variations in the populations across studies. Most importantly some of the topographic factors such as swallowing of tobacco juice and quantity of ST use (cans/pouches) are product specific, therefore, these may not be applicable to the distinct ST product types used by Bangladeshi ST users.

Cotinine is a recommended biomarker to measure dependence among ST users [20]. Past studies of ST dependence have shown inconsistent association between cotinine concentration and dependence measures based on Fagerström Tolerance Questionnaire (FTQ). However, results of the current study found significant association between FTND-ST and cotinine concentration, which are similar to the findings of the past studies of FTND-ST among ST users [21], [22]. Similarly, TTF, which is a brief measure of dependence and has been widely used in cigarette dependence studies, was also positively associated with cotinine concentration. These findings indicate concurrent validity of cotinine concentration as a measure of dependence among ST users.

There are a few limitations of this study including sample selection from urban areas only and potential recall bias. Earlier studies showed notable variation in type of ST use and consumption pattern among urban and rural ST users in Bangladesh [23]. As ST users were selected from urban areas only, it limits the generalizability of the study results. However, to capture the socio-economic dissimilarity among users, we recruited users from both slum and non-slum areas in Dhaka city. The self-reported questionnaire survey might also result in measurement error due to under or over estimation while reporting ST consumption by type and quantity. However, if it had occurred, there would be non-differential misclassification resulting in more conservative findings. Despite a comprehensive evaluation of ST use characteristics, we did not include some of the tobacco use variables such as, dip duration, age of onset of ST use, age regular ST use, initial intent of ST use, and ST use to quit smoking.

High cotinine concentration among ST users and increased prevalence of ST use in Bangladesh [6] indicate that ST products are highly addictive and ST users need adequate support to give up this behaviour. As Bangladesh lacks comprehensive tobacco control programmes there is an urgent need to design and implement effective interventions to create awareness and provide quit support to ST users. Further study on ST consumption among youth, groups at highest risk and cessation success are important to better inform tobacco control initiatives that would effectively reduce burden of harm attributable to ST use. First step to achieve these goals will be to regulate the production and supply of all ST products used in the country.

## Supporting Information

S1 File Smokeless Tobacco Dependence Study- Screener and Main Questionnaire in English. (PDF)

S2 File Smokeless Tobacco Dependence Study- Screener and Main Questionnaire in Bengali. (PDF)

S3 File Data set. (SAV)
