# Supplementary material for: Determinants of Salivary Cotinine among Smokeless Tobacco Users: A Cross-Sectional Survey in Bangladesh
Source: PLoS One. 2016 Aug 9;11(8):e0160211. doi: 10.1371/journal.pone.0160211 (PMC4978394; doi:10.1371/journal.pone.0160211)
Supplement: S1 File — (PDF) [file pone.0160211.s001.pdf]

# ID of respondent:
# Screener - Questions for assessing eligibility

1. How well do you speak Bangla?

   [ ] Very well

   [ ] Well

   [ ] Not well

   [ ] Not at all

[IF ‘NOT WELL’ or ‘NOT AT ALL’, not eligible for main questionnaire]

2. How old are you?

   |__||__| Years

[IF <18 YEARS, not eligible for main questionnaire]

3. Have you used smokeless tobacco (ST) in the past 7 days?

   [ ] Yes

   [ ] No

[IF NO, not eligible for main questionnaire]

4. In the past 6 months, have you used ST on at least 1 day per week?

   [ ] Yes

   [ ] No

[IF NO, not eligible for main questionnaire]

5. For how long have you used ST?

   [ ] [ ] Months

   [ ] [ ] Years

[IF < 1 YEAR, not eligible for main questionnaire]

6. In the past 30 days, have you participated in any type of cessation treatment to help you quit ST such as medication, counseling, or telephone quit-line?

   [ ] Yes

   [ ] No

[IF YES, not eligible for main questionnaire]

7. In the past 30 days, have you smoked tobacco (Bidis/Cigarettes/Water pipes/Cigars/Pipes)?

   [ ] Yes

   [ ] No

[IF YES, not eligible for main questionnaire]

8. In the past 30 days, have you used any illegal drugs (such as Heroin, Cocaine, or Cannabis)?

   [ ] Yes

   [ ] No

[IF YES, not eligible for main questionnaire]

9. Have you ever been diagnosed with a psychiatric illness (such as Depression, Anxiety, or Schizophrenia)?

   [ ] Yes

   [ ] No

[IF YES, not eligible for main questionnaire]

# Smokeless Tobacco Dependence Study - Main Questionnaire

## Socio-Demographic Data

1. Please indicate your gender

   [ ] Male

   [ ] Female

2. Are you currently pregnant?

   [ ] Yes

   [ ] No

   [ ] Not Applicable

3. Are you currently breastfeeding?

   [ ] Yes

   [ ] No

   [ ] Not Applicable

4. What is the highest level of education you have completed? (Use Bangladesh Demographic Health Survey Classification)

   [ ] No formal schooling

   [ ] Less than primary school completed

   [ ] Primary school completed

   [ ] Less than secondary school completed

   [ ] Secondary school completed

   [ ] Higher secondary school completed

   [ ] College/University completed

[ ] Post-graduate degree completed

5. Which of the following best describes your main work status? ? (Use Bangladesh Demographic Health Survey Classification)

   [ ] Government employee

   [ ] Non-government employee

   [ ] Self-employed

   [ ] Student

   [ ] Homemaker (Housewife)

   [ ] Retired

   [ ] Unemployed, able to work

   [ ] Unemployed, unable to work

6. Please tell me whether you /your household have the following items:

   [ ] Electricity

   [ ] Flush Toilet

   [ ] Fixed Telephone

   [ ] Cell Telephone

   [ ] Television

   [ ] Radio

   [ ] Refrigerator

   [ ] Car

   [ ] Moped/Scooter/Motorcycle

   [ ] Washing machine

7. What is your Religion? (optional question for Bangladesh)

   [ ] Hindu

[ ] Muslim

[ ] Christian

[ ] Sikh

[ ] Buddhist

[ ] Jain

[ ] Jewish

[ ] Parsi/Zoroastrian

[ ] No religion

[ ] Other, please state ………………………………………………………………

## Smokeless Tobacco Use History

The following questions are about using Smokeless Tobacco (ST) products, such as Tobacco leaf mixture, Betel quid with tobacco, Khaini or tobacco-lime mixture, Gutkha or tobacco-betel nut-catechu mixture, Gul, Mishri, Tobacco paste, Snus, etc.

8. In the past 7 days, on how many days did you use ST?

   |__| Days

9. In the past 7 days, on the days that you used ST, how many times per day did you use ST?

   |__||__|Times

10. How old were you when you first used ST?

    |__||__| Years

11. Have you ever used ST daily for 6 months or more?

    [ ] Yes

[ ] No

12. Think of the time in your life when you used THE MOST amount of ST.During that time, on how many days per week did you use ST?

|__| Days per week

13. Think of the time in your life when you used THE MOST amount of ST. During that time, on the days that you used ST, how many times per day did you use ST?

|__||__| Times per day

14. Do you currently use ST on a daily (or) less than daily basis?

[ ] Daily

[ ] Less than daily

a. [IF DAILY:] How long have you been using ST daily?

|__||__| Weeks

|__||__| Months

|__||__| Years

15. Are you currently trying to cut down on your ST use?

[ ] Yes

[ ] No

a. [IF YES:] Why are you currently trying to cut down on your ST use? Check all that apply

[ ] I have been diagnosed with an illness

[ ] The cost of ST use is too high

[ ] I got nagged or people are judging me

[ ] My health

[ ] I have started to feel health effects of ST use

[ ] I want to get control of my life

[ ] I am tired of the smell or taste

[ ] I don't know, I just want to

[ ] Other reason ……………………………………………………………………

16. Which of these ST products have you ever used? Check all that apply

[ ] Tobacco leaf or tobacco leaf mixture (Zarda)

[ ] Betel quid (Pan) with tobacco

[ ] Khaini or tobacco, lime mixture

[ ] Gutka or tobacco, betel-nut & catechu mixture

[ ] Dry tobacco powder (Gul, Mishri)

[ ] Tobacco paste (Creamy snuff, Gudakhu)

[ ] Snus

[ ] Any other form of ST, specify type

17. Which of the following ST products have you used in the past 7 days? For each product, please answer YES (or) NO

a. Tobacco leaf or tobacco leaf mixture

[ ] Yes

[ ] No

i. [IF YES] In the past 7 days, on how many days did you use tobacco leaf or tobacco leaf mixture?

|__||__| Days (Range: 1 - 30)

ii. On the days that you used tobacco leaf or tobacco leaf mixture, how many times on average did you use the producteach day?

|__||__| Times per day (Range: 1 - 99)

b. Betel quid (Pan) with tobacco

[ ] Yes

[ ] No

i. [IF YES] In the past 7 days, on how many days did you use betel quid (Pan) with tobacco?

|__||__| Days (Range: 1 - 30)

ii. On the days that you used betel quid (Pan) with tobacco, how many times on average did you use the product each day?

|__||__| Times per day (Range: 1 - 99)

c. Khaini or tobacco& lime mixture

[ ] Yes

[ ] No

i. [IF YES] In the past 7 days, on how many days did you use Khaini or tobacco& lime mixture?

|__||__| Days (Range: 1 - 30)

ii. On the days that you used Khaini or tobacco& lime mixture, how many times on average did you use the producteach day?

|__||__| Times per day (Range: 1 - 99)

d. Gutka or tobacco, betel-nut & catechu mixture

[ ] Yes

[ ] No

i. [IF YES] In the past 7 days, on how many days did you use Gutka or tobacco, betel-nut & catechu mixture?

|__||__| Days (Range: 1 - 30)

ii. On the days that you used Gutka or tobacco, betel-nut & catechu mixture, how many times on average did you use the producteach day?

|__||__| Times per day (Range: 1 - 99)

e. Dry tobacco powder (Gul, Mishri)

[ ] Yes

[ ] No

i. [IF YES] In the past 7 days, on how many days did you use dry tobacco powder (Gul, Mishri)?

|__||__| Days (Range: 1 - 30)

ii. On the days that you used dry tobacco powder (Gul, Mishri), how many times on average did you use the producteach day?

|__||__| Times per day (Range: 1 - 99)

f. Tobacco paste (Creamy snuff, Gudakhu)

[ ] Yes

[ ] No

i. [IF YES] In the past 7 days, on how many days did you use tobacco paste (Creamy snuff, Gudakhu)?

|__||__| Days (Range: 1 - 30)

ii. On the days that you used tobacco paste (Creamy snuff, Gudakhu), how many times on average did you use the producteach day?

|__||__| Times per day (Range: 1 - 99)

g. Snus

[ ] Yes

[ ] No

i. [IF YES] In the past 7 days, on how many days did you useSnus?

|__||__| Days (Range: 1 - 30)

ii. On the days that you usedSnus, how many times on average did you use the producteach day?

|__||__| Times per day (Range: 1 - 99)

h. ………………………………………………………………………………...

[ ] Yes

[ ] No

i. [IF YES] In the past 7 days, on how many days did you use………………………………………………………………………………………?

|__||__| Days (Range: 1 - 30)

ii. On the days that you used this product, how many times on average did you use iteach day?

|__||__| Times per day (Range: 1 - 99)

i. ………………………………………………………………………………...

[ ] Yes

[ ] No

i. [IF YES] In the past 7 days, on how many days did you use………………………………………………………………………………………?

|__||__| Days (Range: 1 - 30)

ii. On the days that you used this product, how many times on average did you use iteach day?

|__||__| Times per day (Range: 1 - 99)

j. ……………………………………………………

[ ] Yes

[ ] No

i. [IF YES] In the past 7 days, on how many days did you use…………………………………………………………………………………………?

|__||__| Days (Range: 1 - 30)

ii. On the days that you used this product, how many times on average did you use iteach day?

|__||__| Times per day (Range: 1 - 99)

18. In the past 7 days, did you use ST…?

[ ] Mainly when you were with people

[ ] Mainly when you were alone

[ ] As often by yourself as with others

19. How many of your five closest friends use ST?

[ ] None

[ ] One

[ ] Two

[ ] Three

[ ] Four

[ ] All five

20. How many of your five closest relatives use ST?

[ ] None

[ ] One

[ ] Two

[ ] Three

[ ] Four

[ ] All five

21. Among your closest relatives, who all use ST? Check all that apply

    [ ] Parents, Grandparents, Parents-in-law

    [ ]Siblings (including sister- or brother-in-law)

    [ ] Partners (including wife or husband)

    [ ]Children (including son- or daughter-in-law)

    [ ] Any other relatives living in the same household

22. Which statement best describes ST use in your household?

    [ ] ST use is never allowed

    [ ] ST use is allowed during special occasions or when there are visitors

    [ ] ST use is allowed at all times

## Smokeless Tobacco Dependence Scales

***Tobacco Dependence Screener***

23. Have you often had periods of days when you chew/dip a lot more than you intended to?

    ☐Yes ☐ No or Not Applicable

24. Have you ever tried to quit or cut down on tobacco and found you could not?

    ☐Yes ☐ No or Not Applicable

25. Did you crave tobacco after you quit or cut down on it?

    ☐Yes ☐ No or Not Applicable

26. Did you have any of the following problems when you quit or cut down on tobacco: irritation, nervousness, restlessness, trouble concentrating,

headache, drowsiness, upset stomach, heart slow down, increased appetite or body weight, hand-shakes, depression?

☐Yes ☐ No or Not Applicable

27. Did you ever start using tobacco again to keep from having such problems?

☐Yes ☐ No or Not Applicable

28. Have you ever continued to chew/dip when you had a serious illness that you knew made it unwise to use tobacco?

☐Yes ☐ No or Not Applicable

29. Did you continue to use tobacco after you knew that it caused you health problems?

☐Yes ☐ No or Not Applicable

30. Did you continue to use tobacco after you knew that it caused you mental problems?

☐Yes ☐ No or Not Applicable

31. Have you ever felt like you were dependent on tobacco?

☐Yes ☐ No or Not Applicable

32. Have you ever given up work or social activities so you could use tobacco?

☐Yes ☐ No or Not Applicable

***FagerströmTest for Nicotine Dependence - Smokeless Tobacco (FTND-ST)***

33. How soon after you wake up do you place your first dip?

☐Within 5 min ☐6–30 min ☐31–60 min ☐After 60 min

34. How often do you intentionally swallow tobacco juice?

☐ Always ☐ Sometimes ☐ Never

35. Which chew would you hate to give up most?

☐ The first one in the morning ☐ Any other

36. How many cans/pouches per week do you use?

☐ More than 3 ☐ 2–3 ☐ 1

37. Do you chew more frequently during the first hours after awakening than during the rest of the day?

☐ Yes ☐ No

38. Do you chew if you are so ill that you are in bed most of the day?

☐ Yes ☐ No

## *Oklahoma Scale for Smokeless Tobacco Dependence (OSSTD)*

Please rate your level of agreement for each of the statement using the following scale:

| 1 | 2 | 3 | 4 | 5 | 6 | 7 |
|---|---|---|---|---|---|---|
| Not true of me at all | | | | | | Extremely true of me |

CIRCLE ONE NUMBER FOR EACH ITEM

| | | | | | | | | |
|---|---|---|---|---|---|---|---|---|
| 39. | Chew/dip controls me | 1 | 2 | 3 | 4 | 5 | 6 | 7 |
| 40. | Chewing/dipping improves my mood | 1 | 2 | 3 | 4 | 5 | 6 | 7 |
| 41. | Very few things give me pleasure each day like chewing/dipping | 1 | 2 | 3 | 4 | 5 | 6 | 7 |
| 42. | It's hard to ignore an urge to chew/dip | 1 | 2 | 3 | 4 | 5 | 6 | 7 |
| 43. | I chew/dip when I really need to concentrate | 1 | 2 | 3 | 4 | 5 | 6 | 7 |

| | | | | | | | | |
|---|---|---|---|---|---|---|---|---|
| 44. | I rely upon chewing/dipping to control my hunger and eating | 1 | 2 | 3 | 4 | 5 | 6 | 7 |
| 45. | Chew/dip keep me company, like a close friend | 1 | 2 | 3 | 4 | 5 | 6 | 7 |
| 46. | There are particular sights and smells that trigger strong urges to chew/dip | 1 | 2 | 3 | 4 | 5 | 6 | 7 |
| 47. | Chewing/dipping helps me stay focused | 1 | 2 | 3 | 4 | 5 | 6 | 7 |
| 48. | I frequently crave chew/dip | 1 | 2 | 3 | 4 | 5 | 6 | 7 |
| 49. | Weight control is a major reason that I chew/dip | 1 | 2 | 3 | 4 | 5 | 6 | 7 |
| 50. | I'm really hooked on chew/dip | 1 | 2 | 3 | 4 | 5 | 6 | 7 |
| 51. | I find myself reaching for chew/dip without thinking about it | 1 | 2 | 3 | 4 | 5 | 6 | 7 |
| 52. | I crave chew/dip at certain times of the day | 1 | 2 | 3 | 4 | 5 | 6 | 7 |
| 53. | I would feel alone without my chew/dip | 1 | 2 | 3 | 4 | 5 | 6 | 7 |
| 54. | Other chewers/ dippers would consider me a heavy chewer/dipper | 1 | 2 | 3 | 4 | 5 | 6 | 7 |
| 55. | Some things are very hard to do without chewing/dipping | 1 | 2 | 3 | 4 | 5 | 6 | 7 |
| 56. | I chew/dip within the first 30 minutes of awakening in the morning | 1 | 2 | 3 | 4 | 5 | 6 | 7 |
| 57. | Sometimes I am not aware that I am chewing/dipping | 1 | 2 | 3 | 4 | 5 | 6 | 7 |
| 58. | Chewing/dipping helps think better | 1 | 2 | 3 | 4 | 5 | 6 | 7 |
| 59. | Chewing/dipping really helps me feel better if I've been feeling down | 1 | 2 | 3 | 4 | 5 | 6 | 7 |
| 60. | Chewing/dipping makes me feel good | 1 | 2 | 3 | 4 | 5 | 6 | 7 |
| 61. | Chewing/dipping keeps me from over eating | 1 | 2 | 3 | 4 | 5 | 6 | 7 |

62. Please rate your addiction to ST using the following scale

| 1 | 2 | 3 | 4 | 5 | 6 | 7 |
|---|---|---|---|---|---|---|
| I am not addicted to ST at all | | | | | | I am extremely addicted to ST |

## Smokeless Tobacco - Buying and Carrying

63. Do you usually carry ST with you?

[ ] Yes

[ ] No

64. The last time you bought ST for yourself, how many of did you buy? [RECORD NUMBER AND CHECK UNIT BELOW]

|__||__||__|

[ ] Packets

[ ] Cans

[ ] Other, specify ……………………………………………………………………….

65. For how long was this buy meant to last?

|__| Hours

|__| Days

|__| Weeks

|__| Months

66. In total, how much money did you pay for this purchase? [IF DON'T KNOW, ENTER 999]

|__||__||__| Taka/Pound

67. The last time you purchased ST products for yourself, where did you buy them?

[ ] Kiosk

[ ] Street vendor

[ ] Store

[ ] Internet

[ ] Outside the country

[ ] From another person

[ ] Other, specify ……………………………………………………………………..

68. How much of the time have you felt the urge to use ST in the past 24 hours?

| Not at all | A little of the time | Some of the time | A lot of the time | Almost all the time | All the time |
|---|---|---|---|---|---|
| 0 | 1 | 2 | 3 | 4 | 5 |

69. How strong have the urges been?

| No urges | Slight | Moderate | Strong | Very strong | Extremely strong |
|---|---|---|---|---|---|
| 0 | 1 | 2 | 3 | 4 | 5 |

70. During the past 12 months, have you tried to quit using ST completely?

[ ] Yes

[ ] No

a. [IF YES:]During the past 12 months, how many times have you stopped using ST for one day or longer <u>because you were trying to quit</u>?

|__||__||__| Number of times

b. During the past 12 months what is the longest length of time you stopped using ST <u>because you were trying to quit</u>?

|__||__| Hours

|__||__| Days

|__||__| Weeks

|__||__| Months

c. Which of these statements best describes how your most recent quit attempt started:

[ ] I did not plan the quit attempt in advance, I just did it

[ ] I planned the quit attempt for later the same day

[ ] I planned the quit attempt the day beforehand

[ ] I planned the quit attempt a few days beforehand

[ ] I planned the quit attempt a few weeks beforehand

[ ] I planned the quit attempt a few months beforehand

71. Have you ever used any of the following methods to help you stop ST use? Check all that apply

[ ] Counseling, including at a tobacco cessation clinic

[ ] Nicotine replacement therapy, such as the patch or gum

[ ] Other prescription medications, for example Bupropion

[ ] Traditional medicines, for example Ayurvedic, Homeopathic, Unani

[ ] A quit line or a ST telephone support line

[ ] I quit on my own, did not use anything

[ ] I have never tried to quit using ST

[ ] Other, please describe …………………………………………………………

72. What best describes your intentions to stop using ST completely? Would you say you…?

[ ] Never expect to quit

[ ] May quit in the future, but not in the next 6 months

[ ] Will quit in the next 6 months

[ ] Will quit in the next 30 days

73. During the past 12 months, how many times did you visit a doctor or health care provider to be seen for a routine examination or an illness or injury?

|__||__||__| Number of times

74. During any visit to a doctor or health care provider in the past 12 months, were you asked if you use ST?

[ ] Yes

[ ] No

75. During any visit to a doctor or health care provider in the past 12 months, were you advised to stop using ST?

[ ] Yes

[ ] No

76. During any visit to a doctor or health care provider in the past 12 months, were you given assistance to stop using ST, such as specific advice on how to quit ST or prescribed medication?

[ ] Yes

[ ] No

77. During any visit to a doctor or health care provider in the past 12 months, did the doctor or health care provider arrange follow-up with their office about quitting ST or refer you to a tobacco cessation program?

[ ] Yes

[ ] No

78. Please choose the number of the response that best describes your opinion: If you continue to use ST, how likely do you think it is that you will develop oral cancer?

| No chance | Very unlikely | Unlikely | Moderate chance | Likely | Very likely | Certain to happen |
|---|---|---|---|---|---|---|
| 1 | 2 | 3 | 4 | 5 | 6 | 7 |

79. Please choose the number of the response that best describes your opinion: If you continue to use ST, how likely do you think it is that you will develop heart disease?

| No chance 1 | Very unlikely 2 | Unlikely 3 | Moderate chance 4 | Likely 5 | Very likely 6 | Certain to happen 7 |
|---|---|---|---|---|---|---|

80. In your opinion, compared to smoking, using ST has …

    [ ] More health risks

    [ ] Less health risks

    [ ] Same health risks

81. In the last year, how often did you try to limit your ST use to decrease your health risks?

    [ ] Never

    [ ] Rarely

    [ ] Sometimes

    [ ] Often

    [ ] Always

## Past Smoking History

I would now like to ask you some questions about your past smoking tobacco, including *bidis*, cigarettes, cigars, cheroots, rolled cigarettes, tobacco rolled in maize leaf and newspaper, water pipe, pipes, chillum, chutta. *Please do not answer about ST at this time*.

82. Have you smoked at least 100 cigarettes in your entire life time?

    [ ] Yes

    [ ] No

    [IF NO, Skip section]

83. How old were you when you first started smoking tobacco?

|__||__| Years

84. Have you ever smoked daily for 6 months or more?

[ ] Yes

[ ] No

85. Think of the time in your life when you SMOKED THE MOST. During that time, on how many days per week did you smoke?

|__| Days

86. Think of the time in your life when you SMOKED THE MOST. During that time, on the days that you smoked, how many times /cigarettes did you smoke per day?

|__||__| Times /Cigarettes per day

87. Think of the time in your life when you SMOKED THE MOST. During that time, which of these products did you commonly use? Check all that apply

[ ] Manufactured cigarette

[ ] Hand-rolled tobacco in paper or leaf

[ ] Bidi

[ ] Cigar

[ ] Cheroot

[ ] Cigarillo

[ ] Pipe

[ ] Water pipe

[ ] Any other form, specify type …………………………………………………

88. How long has it been since you stopped smoking?

|__||__| Months

|__||__| Years

89. Did you use any of the following methods to help you quit smoking? Check all that apply

[ ] Counseling, including at a tobacco cessation clinic

[ ] Nicotine replacement therapy, such as the patch or gum

[ ] Other prescription medications, for example Bupropion

[ ] Traditional medicines, for example Ayurvedic, Homeopathic, Unani

[ ] A quit line or a smoking telephone support line

[ ] Switching to smokeless tobacco

[ ] I quit on my own, did not use anything

[ ] Other, please describe ……………………………………………………………..

90. Why did you stop smoking?

[ ] I was diagnosed with an illness

[ ] The cost of smoking was too high

[ ] I got nagged or people were judging me

[ ] I started to feel health effects of smoking

[ ] My health

[ ] I was tired of feeling out of breath

[ ] I wanted to get control of my life

[ ] I was tired of the smell or taste

[ ] I don't know, I just did

[ ] Smoking bans or restrictions at home or public places

[ ] Other reasons ……………………………………………………………………….

## Health Behaviours

Now I would like to ask you some questions about your general health condition. Has a doctor or health care provider ever told you that you had any of the following?

91. Heart attack, also called myocardial infarction?

    [ ] Yes

    [ ] No

    [ ] Don't know

92. Angina or coronary heart disease?

    [ ] Yes

    [ ] No

    [ ] Don't know

93. Hypertension or raised blood pressure?

    [ ] Yes

    [ ] No

    [ ] Don't know

94. Cancer?

    [ ] Yes

    [ ] No

    [ ] Don't know

95. Asthma?

    [ ] Yes

    [ ] No

    [ ] Don't know

96. Teeth and gum problems?

[ ] Yes

[ ] No

[ ] Don’t know

97. During the past month, other than your regular job, on how many days did you participate in any physical activities or exercises?

|__||__|Days per month

a. [IF > 0:] When you took part in this activity, for how many minutes or hours did you usually keep at it?

|__||__| Minutes

|__||__| Hours

98. Over the past 7 days, on average how many servings of fruit did you eat per day

|__||__| Servings

99. Over the past 7 days, on average how many servings of vegetables did you eat per day

|__||__| Servings

100. In general, would you say your health is…?

[ ] Excellent

[ ] Very Good

[ ] Good

[ ] Fair

[ ] Poor

# Smokeless tobacco (ST) dependence study - Questionnaire Construct & Reference

## Screener *(Created for ST dependence study)*

| Construct | Instrument | # of Items | Survey # |
|---|---|---|---|
| Language | | 1 | 1 |
| Age | | 1 | 2 |
| ST use screener: In the past week, regular use & length of time | | 3 | 3 - 5 |
| ST cessation assistance | | 1 | 6 |
| Smoking screener: In the last 30 days | | 2 | 7 - 8 |
| Any drug use in the last 30 days | | 1 | 8 |
| History of mental illness | | 1 | 9 |

## Main Questionnaire

| Construct | Instrument | # of Items | Survey # |
|---|---|---|---|
| **Socio-demographic** | | | |
| Age | Screener | 1 | 2 (Screener) |
| Gender, Pregnancy, Breast-feeding | | 3 | 1 - 3 |
| Education, Employment, Assets | GATS - India[i] | 3 | 4 - 6 |
| Religion (optional for Bangladesh) | Census of India[ii] | 1 | 7 |

| | | | |
|---|---|---|---|
| **ST use history** | | | |
| ST use in the past week | | 2 | 8, 9 |
| Age of 1st use | OSSTD study[iii] | 1 | 10 |
| Daily / Quantifying maximum use | TUS - CPS[iv] (modified for ST users)<br>OSSTD study[v] | 5 | 11 - 13<br>14, 14a |
| Reducing ST use, reasons | Modified from Nondaily Cigarette Smoking Study[vi] | 2 | 15, 15a |
| ST products | Modified using GATS - India[vii] | 11 | 16, 17 (a - j) |
| Social use, use by friends and family members | Created for study + 1 item from NYTS (modified for ST) | 5 | 18 - 22 |
| **ST dependence** | | | |
| Tobacco Dependence Screener[viii] | Modified for ST use[ix] | 10 | 23 - 32 |
| Fagerström Test for Nicotine Dependence - Smokeless Tobacco (FTND-ST)[x] | | 6 | 33 - 38 |
| Oklahoma Scale for Smokeless Tobacco Dependence (OSSTD) | OSSTD study[xi] | 23 | 39 - 61 |
| Self-rated addiction | 1 item from CDS-12[xii] (modified for ST) | 1 | 62 |
| **ST behaviors** | | | |
| Carrying ST | California Tobacco Survey[xiii] (modified for ST) | 1 | 63 |
| Buying ST | GATS - India[xiv] + 1 item created for study | 4 | 64 - 67 |
| Urge to use | MPSS[xv] | 2 | 68, 69 |
| Past year quit attempts | Modified from Nondaily Cigarette Smoking Study[xvi] | 3 | 70, 70a - b |
| Planned quit attempts | West &Sohal, 2006[xvii] | 1 | 70c |
| Use of assistance to quit ST | Modified from GATS - India[xviii] | 1 | 71 |
| Quit intention | Prochaska, Diclemente, 1991[xix] (modified for ST) | 1 | 72 |
| Physician advise to quit | California Tobacco Survey[xx] (modified for ST) | 5 | 73 - 77 |

| | | | |
|---|---|---|---|
| **Health** | | | |
| Perceived vulnerability | Borrelli et al[xxi] (modified for ST) | 2 | 78, 79 |
| Harm reduction, Smoking vs. ST | 1 item from KIS-II[xxii] | 2 | 80, 81 |
| Past MI, Angina, Hypertension etc. | Adapted from BRFSS 2011[xxiii] | 6 | 91 - 96 |
| Exercise, diet | BRFSS 2011[xxiv] | 4 | 97, 97a - 99 |
| Health question | 1 item from SF-36[xxv] | 1 | 100 |
| **Smoking history/variables** | | | |
| Lifetime 100 cigarettes | CDC[xxvi] | 1 | 82 |
| Age of 1st use | | 1 | 83 |
| Daily / Quantifying maximum use | TUS - CPS[xxvii] | 3 | 84 - 86 |
| Smoked tobacco products | | 1 | 87 |
| Quit history, Use of assistance to quit smoking | Modified from GATS - India[xxviii] | 2 | 88, 89 |
| Reasons to quit | Ahluwalia et al.[xxix] | 1 | 90 |

## ***Reference:***

[i] WHO 2010; GATS (Global Adult Tobacco Survey);*India: core questionnaire with optional questions*
[ii] Census of India 2011; http://www.censusindia.gov.in/
[iii] MUSHTAQ, N., BEEBE, L. A., VESELY, S. K. & NEAS, B. R. 2013; A multiple motive/multi-dimensional approach to measure smokeless tobacco dependence. *Addictive behaviours*
[iv] US Department of Commerce, Census Bureau 2012; National Cancer Institute-sponsored Tobacco Use Supplement to the Current Population Survey (2010-11): http://appliedresearch.cancer.gov/tus-cps/info.html
[v] MUSHTAQ, N., BEEBE, L. A., VESELY, S. K. & NEAS, B. R. 2013; A multiple motive/multi-dimensional approach to measure smokeless tobacco dependence. *Addictive behaviours*
[vi] AHLUWALIA 2011; Pfizer GRAND Program - Factors Influencing Nondaily Cigarette Smoking and Cessation Conduct nine focus groups to: (Aim 1) and to (Aim 2) conduct an online survey of 2,400 triethnic smokers.
[vii] WHO 2010; GATS (Global Adult Tobacco Survey);*India: core questionnaire with optional questions*
[viii] KAWAKAMI, N., TAKATSUKA, N., INABA, S. & SHIMIZU, H. 1999; Development of a screening questionnaire for tobacco/nicotine dependence according to Icd-10,Dsm-III-r, and Dsm-IV;*Addictive behaviours,* 24**,** 155-166
[ix] MUSHTAQ, N., BEEBE, L. A. & VESELY, S. K. 2012; Determinants of Salivary Cotinine Concentrations among Smokeless Tobacco Users;*Nicotine & Tobacco Research,* 14**,** 1229-1234
[x] EBBERT, J. O., PATTEN, C. A. & SCHROEDER, D. R. 2006; The fagerström test for nicotine dependence-smokeless tobacco (FTND-ST);*Addictive behaviours,* 31**,** 1716-1721
[xi] MUSHTAQ, N., BEEBE, L. A., VESELY, S. K. & NEAS, B. R. 2013; A multiple motive/multi-dimensional approach to measure smokeless tobacco dependence. *Addictive behaviours*

[xii]ETTER, J.-F., LE HOUEZEC, J. & PERNEGER, T. V. 2003; A self-administered questionnaire to measure dependence on cigarettes: the cigarette dependence scale;*Neuropsychopharmacology,* 28**,** 359-370
[xiii] California Tobacco Surveys (CTS) 2008; http://libraries.ucsd.edu/locations/sshl/data-gov-info-gis/ssds/guides/tobacco-surveys.html
[xiv]WHO 2010; GATS (Global Adult Tobacco Survey);*India: core questionnaire with optional questions*
[xv] Mood and Physical Symptoms Scale (MPSS); NCSCT: http://www.ncsct.co.uk/publication_mood-and-physical-symptoms-scale%20.php
[xvi] AHLUWALIA 2011; Pfizer GRAND Program - Factors Influencing Nondaily Cigarette Smoking and Cessation Conduct nine focus groups to: (Aim 1) and to (Aim 2) conduct an online survey of 2,400 triethnic smokers.
[xvii]WEST, R. & SOHAL, T. 2006; "Catastrophic" pathways to smoking cessation: findings from national survey. *Bmj,* 332**,** 458-460
[xviii]WHO 2010; GATS (Global Adult Tobacco Survey);*India: core questionnaire with optional questions*
[xix]DICLEMENTE, C. C., PROCHASKA, J. O., FAIRHURST, S. K., VELICER, W. F., VELASQUEZ, M. M. & ROSSI, J. S. 1991; The process of smoking cessation: an analysis of precontemplation, contemplation, and preparation stages of change;*Journal of consulting and clinical psychology,* 59**,** 295
[xx] California Tobacco Surveys (CTS) 2008; http://libraries.ucsd.edu/locations/sshl/data-gov-info-gis/ssds/guides/tobacco-surveys.html
[xxi]BORRELLI, B., HAYES, R. B., DUNSIGER, S. & FAVA, J. L. 2010; Risk perception and smoking behavior in medically ill smokers: a prospective study. *Addiction,* 105**,** 1100-1108.
[xxii]AHLUWALIA, J. S., OKUYEMI, K., NOLLEN, N., CHOI, W. S., KAUR, H., PULVERS, K. & MAYO, M. S. 2006; The effects of nicotine gum and counseling among African American light smokers: a 2× 2 factorial design. *Addiction,* 101**,** 883-891.
[xxiii]CDC 2011; BRFSS: Turning information into health; 2011
[xxiv]CDC 2011; BRFSS: Turning information into health; 2011
[xxv]Ware, J. E.,Kosinski, M., Dewey, J. E. &Gandek, B. 2000;*SF-36 health survey: manual and interpretation guide*, Quality Metric Inc
[xxvi] CDC Adult Tobacco Use Information; http://www.cdc.gov/nchs/nhis/tobacco/tobacco_glossary.htm
[xxvii]US Department of Commerce, Census Bureau 2012; National Cancer Institute-sponsored Tobacco Use Supplement to the Current Population Survey (2010-11): http://appliedresearch.cancer.gov/tus-cps/info.html
[xxviii]WHO 2010; GATS (Global Adult Tobacco Survey);*India: core questionnaire with optional questions*
[xxix]AHLUWALIA, J. S., RESNICOW, K. & CLARK, W. S. 1997; Knowledge about smoking, reasons for smoking, and reasons for wishing to quit in inner-city African Americans. *Ethnicity & disease,* 8**,** 385-393
