# Supplementary material for: Determinants of Salivary Cotinine among Smokeless Tobacco Users: A Cross-Sectional Survey in Bangladesh
Source: PLoS One. 2016 Aug 9;11(8):e0160211. doi: 10.1371/journal.pone.0160211 (PMC4978394; doi:10.1371/journal.pone.0160211)
Supplement: S2 File — (PDF) [file pone.0160211.s002.pdf]

উত্তরদাতার আইডি:

Screener-যোগ্যতা পরিমাপের প্রশ্নসমূহ

১. আপনি কত ভালো বাংলা বলতে পারেন?

[ ] খুব ভালো

[ ] ভালো

[ ] ভালো না

[ ] একেবারেই না

[যদি ভালো না অথবা একেবারেই না হয় তবে প্রধান প্রশ্নাবলীর জন্য উপযুক্ত নয়]

২. আপনার বয়স কত?

|__||__|বছর

[যদি ১৮ বছরের কম হয় তবে প্রধান প্রশ্নাবলীর জন্য উপযুক্ত নয়]

৩. আপনি কি বিগত ৭ দিনে ধোঁয়াহীন তামাক ব্যবহার করেছেন?

[ ] হ্যাঁ

[ ] না

[যদি না হয় তবে প্রধান প্রশ্নাবলীর জন্য উপযুক্ত নয়]

৪. বিগত ৬ মাসে আপনি কি প্রতি সপ্তাহে অন্তত ১ দিন তামাক ব্যবহার করেছেন?

[ ] হ্যাঁ

[ ] না

[যদি না হয় তবে প্রধান প্রশ্নাবলীর জন্য উপযুক্ত নয়]

৫. আপনি কত সময় ধরে ধোঁয়াহীন তামাক ব্যবহার করছেন?

|__||__|মাস

|__||__|বছর

[যদি ১ বছরের কম হয় তবে প্রধান প্রশ্নাবলীর জন্য উপযুক্ত নয়]

৬. বিগত ৩০ দিনে ধোঁয়াহীন তামাক ব্যবহার ছাড়ার জন্য আপনি কি কোন ধরনের চিকিৎসা নিয়েছেন, যেমন ঔষধগ্রহন, পরামর্শ অথবা টেলিফোন এ সাহায্য?

[ ] হ্যাঁ

[ ] না

[যদি হ্যাঁ হয় তবে প্রধান প্রশ্নাবলীর জন্য উপযুক্ত নয়]

৭. বিগত ৩০ দিনে আপনি কি তামাক গ্রহন করেছেন (বিড়ি/সিগারেট/হুঁকা/চুরুট/পাইপ)?

[ ] হ্যাঁ

[ ] না

[যদি হ্যাঁ হয় তবে প্রধান প্রশ্নাবলীর জন্য উপযুক্ত নয়]

৮. বিগত ৩০ দিনে আপনি কি অবৈধ তামাক গ্রহণ করেছেন (যেমন- হেরোইন, কোকেন, ভাং)?

[ ] হ্যাঁ

[ ] না

[যদি হ্যাঁ হয় তবে প্রধান প্রশ্নাবলীর জন্য উপযুক্ত নয়]

৯. আপনার কি কখনো কোন মানসিক রোগ নির্ণীত হয়েছে (যেমন-হতাশা, ভীতি, সিজোফ্রেনিয়া)?

[ ] হ্যাঁ

[ ] না

[যদি হ্যাঁ হয় তবে প্রধান প্রশ্নাবলীর জন্য উপযুক্ত নয়]

ধোঁয়াহীন তামাক নির্ভরতার গবেষণা- প্রধান প্রশ্নাবলীসমূহ

সামাজিক-জনতাত্ত্বিক তথ্য

১. আপনার লিঙ্গ বলুন

[ ] পুরুষ

[ ] নারী

২. আপনি কি বর্তমানে গর্ভবতী? (শুধু মাত্র মহিলাদের ক্ষেত্রে প্রযোজ্য)

[ ] হ্যাঁ

[ ] না

[ ] প্রযোজ্য নয়

৩. আপনি কি বর্তমানে শিশুকে বুকের দুধ পান করাচ্ছেন? (শুধু মাত্র মহিলাদের ক্ষেত্রে প্রযোজ্য)

[ ] হ্যাঁ

[ ] না

[ ] প্রযোজ্য নয়

৪. আপনি কতদূর পড়াশুনা করেছেন? (বাংলাদেশ ডেমোগ্রাফিক হেল্থ সার্ভে ক্লাসিফিকেশন ব্যবহার করুন)

[ ] কোন প্রাতিষ্ঠানিক শিক্ষা নেই

[ ] প্রাইমারী স্কুল পাস-এর থেকে কম

[ ] প্রাইমারী স্কুল পাস

[ ] সেকেন্ডারি স্কুল পাস-এর থেকে কম

[ ] সেকেন্ডারি স্কুল পাস

[ ] উচ্চ মাধ্যমিক পাস

[ ] কলেজ/বিশ্ববিদ্যালয় পাস

[ ] স্নাতকোত্তর ডিগ্রি পাস

৫. নিচের কোনটি আপনার পেশাকে ভালভাবে বর্ণনা করে? (বাংলাদেশ ডেমোগ্রাফিক হেল্থ সার্ভে ক্লাসিফিকেশন ব্যবহার করুন)

[ ] সরকারি চাকুরীজীবী

[ ] বেসরকারি চাকুরীজীবী

[ ] স্ব-নিযুক্ত

[ ] ছাত্র

[ ] গৃহিনী

[ ] অবসরপ্রাপ্ত

[ ] বেকার (কাজ করার উপযুক্ত)

[ ] বেকার (কাজ করার অনুপযুক্ত)

৬. দয়া করে বলুন আপনার/আপনার পরিবারের নিচের কোন জিসিগুলো আছে-

[ ] বিদ্যুৎ

[ ] ফ্লাশ টয়লেট

[ ] ফিক্সড টেলিফোন

[ ] মোবাইল ফোন

[ ] টেলিভিশন

[ ] রেডিও

[ ] ফ্রিজ

[ ] গাড়ি

[ ] স্কুটার/ মোটরসাইকেল

[ ] ওয়াশিং মেশিন

৭. আপনার ধর্ম কি

[ ] হিন্দু

[ ] মুসলিম

[ ] খ্রিস্টান

[ ] শিখ

[ ] বৌদ্ধ

[ ] জৈন

[ ] ইহুদী

[ ] পার্সি/জরথুস্টীয়

[ ] কোন ধর্ম নেই

[ ] অন্যান্য, বর্ণনা করুন ………………………………………………………………………

ধোঁয়াহীন তামাক ব্যবহারে ইতিহাস

নিচের প্রশ্নগুলো ধোঁয়াহীন তামাক ব্যবহার সম্পর্কে, যেমন তামাক পাতা মিশ্রন, পানের সাথে তামাক, খৈনি অথবা তামাক চুন মিশ্রণ, গুটকা অথবা তামাক-সুপারী-খয়ের মিশ্রণ, গুল, মিশ্রী, তামাক পেস্ট, নস্যি ইত্যাদি।

৮. বিগত ৭ দিনের মধ্যে আপনি কতদিন ধোঁয়াহীন তামাক ব্যবহার করেছেন?

|__|দিন।

৯. বিগত ৭ দিনে আপনি যেসব দিন ধোঁয়াহীন তামাক গ্রহণ করেছেন, সেসব দিন আপনি দৈনিক কতবার তামাক গ্রহণ করেছেন?

|__||__|বার

১০. যখন আপনি প্রথম ধোঁয়াহীন তামাক গ্রহণ করেছিলেন তখন আপনার বয়স কত ছিল?

|__||__| বছর

১১. আপনি কি কখনো ৬ মাস বা তার বেশি দৈনিক ধোঁয়াহীন তামাক গ্রহণ করেছেন?

|__| হ্যাঁ

|__|না

১২. আপনার জীবনের সেই সময়ের কথা স্মরণ করুন যখন আপনি ***সবচেয়ে বেশি*** ধোঁয়াহীন তামাক গ্রহণ করতেন। সে সময় আপনি প্রতি সপ্তাহে কতদিন ধোঁয়াহীন তামাক গ্রহন করতেন?

|__|দিন প্রতি সপ্তাহে

১৩. আপনার জীবনের সেই সময়ের কথা স্মরণ করুন যখন আপনি ***সবচেয়ে বেশি*** ধোঁয়াহীন তামাক গ্রহণ করতেন। তখন যেসব দিন আপনি ধোঁয়াহীন তামাক গ্রহণ করতেন, সেসব দিনে দৈনিক কতবার ধোঁয়াহীন তামাক গ্রহণ করতেন?

|__|বার প্রতিদিন

১৪. আপনি বর্তমানে ধোঁয়াহীন তামাক দৈনিক (বা) তার থেকে কম ব্যবহার করেন?

|__|দৈনিক

|__|দৈনিকের চেয়ে কম

[যদি দৈনিক] আপনি কতদিন ধরে দৈনিক ধোঁয়াহীন তামাক গ্রহণ করছেন?

|__||__|সপ্তাহ

|__||__|মাস

|__||__|বছর

১৫. আপনি কি বর্তমানে আপনার ধোঁয়াহীন তামাক গ্রহণ কমাতে চেষ্টা করেছেন?

[ ] হ্যাঁ

[ ] না

[যদি হ্যাঁ হয়] কেন আপনি তামাক গ্রহণ কমাতে চাচ্ছেন? যা যা প্রযোজ্য, সবগুলো বলুন

[ ] আমার রোগ ধরা পড়েছে।

[ ] ধোঁয়াহীন তামাক গ্রহনের খরচ খুব বেশি।

[ ] সবাই বারবার বলছে অথবা আমার ব্যাপারে মতামত দিচ্ছে।

[ ] আমার স্বাস্থ্য

[ ] ধোঁয়াহীন তামাক ব্যবহারের ফলে স্বাস্থ্যে এর প্রভাব বুঝতে পারছি।

[ ] আমি আমার জীবনকে নিয়ন্ত্রণে আনতে চাই।

[ ] আমি এর গন্ধ বা স্বাদে ক্লান্ত।

[ ] আমি জানিনা, শুধু ছাড়তে চাই।

[ ] অন্যান্য কারণ ....

১৬. আপনি কোন ধরনের তামাকজাত পণ্যগুলো এখন পর্যন্ত ব্যবহার করেছেন? যেগুলো প্রযোজ্য সবগুলো চিহ্নিত করুন

[ ] তামাক পাতা বা তামাক পাতার মিশ্রণ (জর্দা)

[ ] পানের সাথে তামাক

[ ] খৈনি অথবা তামাক, চুন মিশ্রণ

[ ] গুটকা অথবা তামাক, পান-সুপারি ও খয়ের মিশ্রণ

[ ] শুকনা তামাক গুঁড়া (গুল, মিসরী)

[ ] তামাক পেস্ট (Creamy Snuff, গুদাখু)

[ ] নস্যি (Snus)

[ ] অন্য যেকোন ধরনের ধোঁয়াহীন তামাক, বর্ণনা করুন

১৭. নিচের কোন তামাকজাত পণ্যটি আপনি বিগত ৭ দিন ব্যবহার করেছেন? প্রতিটি পণ্যের ক্ষেত্রে হ্যাঁ অথবা না বলুন।

(ক) তামাক পাতা বা তামাক পাতার মিশ্রণ (জর্দা)

[ ] হ্যাঁ

[ ] না

i. [যদি হ্যাঁ হয়] বিগত ৭ দিনে কতদিন আপনি তামাক পাতা বা তামাক পাতার মিশ্রণ (জর্দা) ব্যবহার করেছেন ?
   |__||__|দিন (সীমা ঃ ১-৭)
ii. যেসব দিনে আপনি তামাক পাতা বা তার মিশ্রণ (জর্দা) গ্রহণকরেছেন, সেসব দিন গড়ে প্রতিদিন কতবার আপনি এসব দ্রব্য গ্রহণ করেছেন?
   |__||__|বার দৈনিক (সীমা ঃ ১-৯৯)

(খ) পানের সাথে তামাক

[ ] হ্যাঁ

[ ] না

i. [যদি হ্যাঁ হয়] বিগত ৭ দিনে আপনি কত দিন পানের সাথে তামাক সাথে গ্রহণ করেছেন?
   |__||__|দিন (সীমা ঃ ১-৭)
ii. যেসব দিনে আপনি পানের সাথে তামাক গ্রহণ করেছেন, সে সব দিন গড়ে প্রতিদিন কতবার এসব দ্রব্য গ্রহণকরেছেন?
   |__||__|বার দৈনিক (সীমা ঃ ১-৯৯)

(গ) খৈনি বা তামাক ও চুন মিশ্রণ

[ ] হ্যাঁ

[ ] না

i. [যদি হ্যাঁ হয়] বিগত ৭ দিনে কতদিন আপনি খৈনি বা তামাক ও চুন মিশ্রণ গ্রহণ করেছেন?

|__||__| দিন (সীমা ১-৭)

ii. যেসব দিন আপনি তা গ্রহণ করেছেন, সেসব দিন দৈনিক গড়ে কতবার আপনি এসব দ্রব্য গ্রহণ করেছেন?

|__||__|বার দৈনিক (সীমা ১-৯৯)

(ঘ) গুটকা অথবা তামাক, পান-সুপারি এবং খয়ের মিশ্রণ

[ ] হ্যাঁ

[ ] না

i. [যদি হ্যাঁ হয়] বিগত ৭ দিন আপনি কতদিন গুটকা অথবা তামাক, পান-সুপারি ও খয়ের মিশ্রণ করেছেন?

|__||__|দিন (সীমা ১-৭)

ii. আপনি যেসব দিন তা গ্রহণ করেছেন, সেসব দিন দৈনিক গড়ে কতবার আপনি এসব দ্রব্য গ্রহণ করেছেন?

|__||__|বার প্রতিদিন (সীমা ১-৯৯)

(ঙ) শুকনা তামাক পাউডার (গুল, মিসরী)

[ ] হ্যাঁ

[ ] না

i. [যদি হ্যাঁ হয়] বিগত ৭ দিনে আপনি কতদিন শুকনা তামাক গুঁড়া (গুল, মিসরী) গ্রহণ করেছেন?

|__||__|দিন (সীমা ১-৭)

ii. যেসব দিন আপনি শুকনা তামাক পাউডার (গুল, মিসরী) গ্রহণ করেছেন, সেসব দিন গড়ে কতবার এসব দ্রব্য গ্রহণ করেছে?

|__||__|বার দৈনিক (সীমা ১-৯৯)

(চ) তামাক পেস্ট (creamy snuff, গুদাখু)

[ ] হ্যাঁ

[ ] না

i. [যদি হ্যাঁ হয়] বিগত ৭ দিনে কতদিন আপনি এসব গ্রহণ করেছেন?

|__||__|দিন (সীমা ১-৭)

ii. যেসব দিন আপনি তা গ্রহণ করেছেন, সেসব দিন দৈনিক গড়ে কতবার গ্রহণ করেছেন?

|__||__|বার দৈনিক (সীমা ১-৯৯)

(ছ) নস্যি (Snus)

[ ] হ্যাঁ

[ ] না

i. [যদি হ্যাঁ হয়] বিগত ৭ দিনে কতদিন নস্যি (Snus) গ্রহণ করেছেন?

|__||__|দিন (সীমা ১-৭)

ii. যেসব দিন আপনি তা গ্রহণ করেছেন, সেসব দিন দৈনিক গড়ে কতবার নস্যি (Snus) গ্রহণ করেছেন?

|__||__|বার দৈনিক (সীমা ১-৯৯)

(জ) ....

[ ] হ্যাঁ

[ ] না

i. [যদি হ্যাঁ হয়] বিগত ৭ দিনে কতদিন আপনি ....গ্রহণ করেছেন?

|__||__| দিন (সীমা ১-৭)

ii. যেসব দিন আপনি এসব গ্রহণ করেছেন, সেসব দিন দৈনিক গড়ে কতবার গ্রহণ করেছেন?

|__||__|বার দৈনিক (সীমা ১-৯৯)

(ঝ) ....

[ ] হ্যাঁ

[ ] না

i. [যদি হ্যাঁ হয়] বিগত ৭ দিনে কতদিন আপনি ....গ্রহণ করেছেন?

|__||__|দিন (সীমা ১-৭)

ii. যেসব দিন আপনি এটি গ্রহণ করেছেন, সেসব দিন দৈনিক গড়ে কতবার গ্রহণ করেছেন?

|__||__|বার দৈনিক (সীমা ১-৯৯)

(ঞ) ....

[ ] হ্যাঁ

[ ] না

i. [যদি হ্যাঁ হয়] বিগত ৭ দিনে কতদিন আপনি ....গ্রহণ করেছেন?

|__||__|দিন (সীমা ১-৭)

ii. যেসব দিন আপনি এটি গ্রহণ করেছেন, সেসব দিন দৈনিক গড়ে কতবার গ্রহণ করেছেন?

|__||__|বার দৈনিক (সীমা ১-৯৯)

১৮. বিগত ৭ দিনে আপনি ধোঁয়াহীন তামাক গ্রহণ করেছেন..........?

[ ] প্রধানত যখন মানুষগণের সাথে ছিলেন।

[ ] প্রধানত যখন একা ছিলেন।

[ ] কখনও একাকী বা কখনও অন্য মানুষগণের সাথে।

১৯. আপনার পাঁচজন ঘনিষ্ঠ বন্ধুর মধ্যে কতজন ধোঁয়াহীন তামাক গ্রহণ করেন?

[ ] কেউ না

[ ] একজন

[ ] দুইজন

[ ] তিনজন

[ ] চারজন

[ ] পাঁচজনই

২০. আপনার পাঁচজন ঘনিষ্ঠ আত্মীয়ের মধ্যে কতজন ধোঁয়াহীন তামাক গ্রহণ করেন?

[ ] কেউ না

[ ] একজন

[ ] দুইজন

[ ] তিনজন

[ ] চারজন

[ ] পাঁচজনই

২১. আপনার ঘনিষ্ঠ আত্মীয়ের মধ্যে নিচের কারা ধোঁয়াহীন তামাক ব্যবহার করেন? যেগুলো প্রযোজ্য সবগুলো চিহ্নিত করুন ।

[ ] পিতা-মাতা, দাদা-দাদী, নানা-নানী, শশুড়-শাশুড়ী

[ ] ভাই-বোন (বিবাহ সম্পর্কে ভাই-বোনরাও অন্তর্ভূক্ত হবেন)

[ ] সাথীরা (স্বামী বা স্ত্রীসহ)

[ ] সন্তানরা (বিবাহ সম্পর্কে আত্মীয়রা অন্তর্ভূক্ত হবেন, যেমন- জামাই বা ছেলের বউ ।)

[ ] একই পরিবারের বাস করছে এমন অন্য কোন আত্মীয় ।

২২. নিচের কোনটি আপনার পরিবারে ধোঁয়াহীন তামাক ব্যবহারকে ভালভাবে বর্ণনা করে?

[ ] কখনোই ধোঁয়াহীন তামাক ব্যবহারের অনুমতি দেয়া হয় না ।

[ ] ধোঁয়াহীন তামাক ব্যবহারের অনুমতি দেয়া হয় শুধুমাত্র বিশেষ অনুষ্ঠানে বা যখন অতিথি আসেন ।

[ ] সবসময় ধোঁয়াহীন তামাক ব্যবহারের অনুমতি দেয়া হয় ।

ধোঁয়াহীন তামাক নির্ভরতার স্কেলসমূহঃ

তামাক নির্ভরতা Screener

২৩. মাঝে মাঝে কি দিনে এমন কোন সময়সীমা থাকে যখন আপনি যা চান তার চেয়ে বেশি তামাক চিবান?

☐হ্যাঁ ☐ না বা প্রযোজ্য নয় ।

২৪. কখনও কি এমন হয়েছে যে আপনি তামাক গ্রহণ ছেড়ে দিতে অথবা কমিয়ে দিতে চেষ্টা করেছেন কিন্তু পারেন নি?

☐হ্যাঁ ☐ না বা প্রযোজ্য নয় ।

২৫. তামাক গ্রহণ ছেড়ে দেয়া অথবা কমানোর পরে আপনি কি তা পুনরায় গ্রহণের তীব্র ইচ্ছা অনুভব করেছেন?

☐হ্যাঁ ☐ না বা প্রযোজ্য নয় ।

২৬. তামাক সেবন ছেড়ে দেয়া অথবা কমানোর পরে আপনার কি নিম্নে উল্লেখিত কোন সমস্যা হয়েছিলঃ বিরক্তিভাব, স্নায়ুদূর্বলতা, অস্থিরতা, মনযোগ সমস্যা, মাথাব্যাথা, তন্দ্রাচ্ছন্নতা, পেটের সমস্যা, হৃদস্পন্দনের ধীরগতি, ক্ষুধা বৃদ্ধি বা ওজন বৃদ্ধি, হাত কাঁপা, বিষন্নতা ।

☐হ্যাঁ ☐ না বা প্রযোজ্য নয়।

২৭. এইসব সমস্যা থেকে মুক্ত হওয়ার জন্যে আপনি কি কখনও পুনরায় তামাক গ্রহণ শুরু করেছিলেন?

☐হ্যাঁ ☐ না বা প্রযোজ্য নয়।

২৮. আপনি কি কখনো খুব অসুস্থতার সময় তামাক গ্রহণ উচিত নয় জেনেও তামাক গ্রহণ অব্যাহত রেখেছিলেন?

☐হ্যাঁ ☐ না বা প্রযোজ্য নয়।

২৯. তামাক গ্রহণের কারণে আপনার স্বাস্থ্যগত সমস্যা দেখা দিয়েছিল তা জানা সত্ত্বেও আপনি কি তামাক গ্রহণ অব্যাহত রেখেছিলেন?

☐হ্যাঁ ☐ না বা প্রযোজ্য নয়।

৩০. তামাক গ্রহণের কারণে আপনার মানসিক সমস্যা দেখা দিয়েছিল তা জানা সত্ত্বেও আপনি কি তামাক গ্রহণ অব্যাহত রেখেছিলেন?

☐হ্যাঁ ☐ না বা প্রযোজ্য নয়।

৩১. আপনার কি কখনো মনে হয়েছে যে আপনি তামাকের উপর নির্ভরশীল?

☐হ্যাঁ ☐ না বা প্রযোজ্য নয়।

৩২. আপনি কি তামাক গ্রহণের জন্য কখনো কোন কাজ বা সামাজিক কার্যক্রমে অংশগ্রহণ করা থেকে বিরত ছিলেন?

☐হ্যাঁ ☐ না বা প্রযোজ্য নয়।

The Fagerström Test for Nicotine Dependence-Smokeless Tobacco (FTND-ST)

[ধোঁয়াহীন তামাক নির্ভরতা নির্ণয়ের FTND-ST পরিমাপ]

৩৩. ঘুম থেকে উঠার পর কত দ্রুত সময়ের মাঝে আপনি আপনার প্রথম তামাক গ্রহণ করেন?

☐ ৫ মিনিটের মধ্যে ☐ ৬-৩০ মিনিট ☐ ৩১-৬০ মিনিট ☐ ৬০ মিনিটের পরে

৩৪. আপনি কত ঘন ঘন ইচ্ছাকৃতভাবে তামাক রস গিলে ফেলেন?

☐সবমসময় ☐ মাঝে মাঝে ☐ কখনোই না

৩৫. কোন সময়ে তামাক চিবানো থেকে বিরত থাকাকে আপনি সবচেয়ে অপছন্দ করবেন?

☐সকালের প্রথমটা ☐ অন্য যেকোনটা

৩৬. আপনি প্রতি সপ্তাহে কত কৌটা/ প্যাকেট ব্যবহার করেন?

☐ ৩ এর অধিক ☐ ২-৩ ☐ ১

৩৭. আপনি কি ঘুম থেকে জাগার পর দিনের প্রথম কয়েক ঘন্টার মধ্যে অন্যান্য সময়ের তুলনায় বেশি বার তামাক চিবান?

☐হ্যাঁ ☐ না

৩৮. আপনি যদি অত্যন্ত অসুস্থ্য হয়ে দিনের বেশির ভাগ সময় বিছানায় থাকেন তখনো কি তামাক চিবান?

☐হ্যাঁ ☐ না

Oklahoma Scale for Smokeless Tobacco Dependence (OSSTD)

[ধোঁয়াহীন তামাক নির্ভরতা নির্ণয়ের Oklahama পরিমাপ]

নিম্নলিখিত স্কেল ব্যাবহার করে উল্লিখিত প্রতিটি বিবৃতির ক্ষেত্রে আপনার নিজস্ব সমর্থনের মাত্রা পরিমাপ করুনঃ

১	২	৩	৪	৫	৬	৭
আমার জন্য মোটেই সত্য নয়						আমার জন্য পুরোপুরি সত্য

প্রতিটি বিবৃতির জন্য সবচেয়ে প্রযোজ্য একটি নম্বরে বৃত্ত তৈরী করুনঃ

৩৯.	চর্বণ /রস আস্বাদন আমাকে নিয়ন্ত্রণ করে	১	২	৩	৪	৫	৬	৭
৪০.	চর্বণ /রস আস্বাদন আমার মন প্রফুল্ল করে	১	২	৩	৪	৫	৬	৭
৪১.	প্রতিদিন খুব কম জিনিস আমাকে চর্বণ/রস আস্বাদনের মত আনন্দ দেয়	১	২	৩	৪	৫	৬	৭
৪২.	চর্বণ/রস আস্বাদনের প্রবল ইচ্ছাকে অবজ্ঞা করা কঠিন	১	২	৩	৪	৫	৬	৭
৪৩.	আমি চর্বণ/রস আস্বাদন করি যখন আমার পূর্ণ মনোযোগ প্রয়োজন	১	২	৩	৪	৫	৬	৭
৪৪.	আমি আমার ক্ষুধা এবং আহার নিয়ন্ত্রণ করার জন্য চর্বণ/রস আস্বাদন এর উপর নির্ভর করি	১	২	৩	৪	৫	৬	৭
৪৫.	চর্বণ/রস আস্বাদন আমাকে একজন ঘনিষ্ট বন্ধুর মত সঙ্গ দেয়	১	২	৩	৪	৫	৬	৭
৪৬.	কিছু নির্দিষ্ট দৃশ্য বা গন্ধ আছে যা চবর্ণ/রস আস্বাদনের আগ্রহকে উদ্দীপিত করে	১	২	৩	৪	৫	৬	৭
৪৭.	চর্বণ/রস আস্বাদন আমাকে একাগ্র থাকতে সাহায্য করে	১	২	৩	৪	৫	৬	৭
৪৮.	আমি মাঝে মাঝেই রস আস্বাদন/ চর্বণের জন্য ব্যাকুলতা অনুভব করি	১	২	৩	৪	৫	৬	৭
৪৯.	ওজন নিয়ন্ত্রণ চর্বণ/রস আস্বাদনের একটি প্রধান কারণ	১	২	৩	৪	৫	৬	৭
৫০.	আমি প্রকৃত পক্ষে চর্বণ/রস আস্বাদনে আসক্ত	১	২	৩	৪	৫	৬	৭
৫১.	আমি এ বিষয়ে চিন্তা না করেই নিজেকে চর্বণ/রস আস্বাদনের কাছে পৌঁছে যেতে দেখি	১	২	৩	৪	৫	৬	৭
৫২.	আমি দিনের কিছু নির্দিষ্ট সময়ে চর্বণ/রস আস্বাদনের প্রবল ইচ্ছা বোধ করি	১	২	৩	৪	৫	৬	৭
৫৩.	আমি চর্বণ/রস আস্বাদন ছাড়া একাকীত্ব অনুভব করবো	১	২	৩	৪	৫	৬	৭
৫৪.	অন্যান্য চর্বণকারী/রস আস্বাদনকারীগণ আমাকে অধিক চর্বণকারী/রস আস্বাদনকারী মনে করবেন	১	২	৩	৪	৫	৬	৭

৫৫. কিছু কাজ চর্বণ/রস আস্বাদন ছাড়া করা খুব কঠিন	১	২	৩	৪	৫	৬	৭
৫৬. আমি সকালে ঘুম থেকে ওঠার প্রথম ৩০ মিনিটের মধ্যে চর্বণ/রস আস্বাদন শুরু করে দিই	১	২	৩	৪	৫	৬	৭
৫৭. অনেক সময় আমি নিজের অজান্তে চর্বণ/রস আস্বাদন করতে থাকি	১	২	৩	৪	৫	৬	৭
৫৮. চর্বণ/রস আস্বাদন আমাকে ভালভাবে চিন্তা করতে সাহায্য করে	১	২	৩	৪	৫	৬	৭
৫৯. যদি আমি বিষন্ন থাকি চর্বণ/রস আস্বাদন আমাকে সত্যিকার অর্থে ভাল বোধ করতে সহায়তা করে	১	২	৩	৪	৫	৬	৭
৬০. চর্বণ/রস আস্বাদন আমাকে ভাল বোধ করতে সাহায্য করে	১	২	৩	৪	৫	৬	৭
৬১. চর্বণ/রস আস্বাদন আমাকে অতিরিক্ত খাদ্য গ্রহন থেকে দূরে রাখে	১	২	৩	৪	৫	৬	৭

৬২. আপনার ধোঁয়াহীন তামাক গ্রহনের আসক্তিকে নিম্নের স্কেল অনুযায়ী মাত্রা নির্ধারন করুন।

১	২	৩	৪	৫	৬	৭
আমি ধোঁয়াহীন তামাকে একবাইরেই আসক্ত নই						আমি ধোঁয়াহীন তামাকে প্রবলভাবে আসক্ত

<u>ধোঁয়াহীন তামাক কেনা এবং বহন করা</u>

৬৩. আপনি কি সাধারণত আপনার সাথে ধোঁয়াহীন তামাক বহন করেন?

[ ] হ্যাঁ

[ ] না

৬৪. আপনি যখন আপনার জন্য শেষ ধোঁয়াহীন তামাক কিনেছিলেন, কতগুলো কিনেছিলেন?

(নাম্বারটি লিখুন এবং নিচের ইউনিট লক্ষ্য করুন)

|__||__||__|

|__|প্যাকেট

|__|কৌটা

|__|অন্যান্য, ব্যাখ্যা করুন..

৬৫. আপনি কেনার পর এটা কতক্ষণ পর্যন্ত ব্যবহার করেছিলেন?

|__|ঘণ্টা

|__|দিন

|__|সপ্তাহ

|__|মাস

৬৬. আপনি সর্বমোট কত টাকা খরচ করেছিলেন এটা ক্রয়ের জন্য। (না জানলে ৯৯৯ লিখুন)

|__||__||__| টাকা/পাউন্ড (প্রযোজ্য না হলে কেটে দিন)

৬৭. আপনি শেষ যখন আপনার জন্য তামাক কিনেছিলেন, কোথা থেকে কিনেছিলেন?

[ ] কোন ছোট স্থান থেকে

[ ] রাস্তার বিক্রেতা

[ ] দোকান

[ ] ইন্টারনেট

[ ] দেশের বাইরে থেকে

[ ] অন্য একজনের কাছ থেকে

[ ] অন্যান্য, ব্যাখ্যা করুন...

ধোঁয়াহীন তামাক ঃ আচরণ, স্বাস্থ্যের ঝুঁকি এবং ছেড়ে দেয়ার ইচ্ছা

৬৮. আপনি বিগত ২৪ ঘণ্টায় কতবার তামাক ব্যবহারের প্রবল ইচ্ছা অনুভব করেছেন?

একদমই না	খুব কম বার	মাঝে মাঝে	অনেকবার	মোটামুটি সবসময়	সবসময়
০	১	২	৩	৪	৫

৬৯. আপনার ইচ্ছাটা কতখানি জোরালো ছিল?

একদমই না	সামান্য	মোটামুটি	জোরালো	খুব জোরালো	একেবারে সর্বোচ্চ জোরালো
০	১	২	৩	৪	৫

৭০. বিগত ১২ মাসে আপনি কি ধোঁয়াহীন তামাক ব্যবহার সম্পূর্ণরূপে ছেড়ে দেয়ার চেষ্টা করেছিলেন?

[ ] হ্যাঁ

[ ] না

(ক) [হ্যাঁ হলে] বিগত ১২ মাসে কতবার আপনি একদিন বা তার অধিক সময়ের জন্য তামাক গ্রহণ থেকে বিরত ছিলেন <u>কারণ আপনি তামাক ছেড়ে দিতে চাচ্ছিলেন</u> ?

|__||__||__|বার

(খ) বিগত ১২ মাসে আপনি সর্বোচ্চ কত সময়ের জন্য তামাক গ্রহণ থেকে দূরে ছিলেন <u>কারণ আপনি তামাক গ্রহণ ছেড়ে দিতে চাচ্ছিলেন</u>?

|__||__|ঘণ্টা

|__||__|দিন

|__||__|সপ্তাহ

|__||__|মাস

(গ) নিচের কোন বক্তব্যটি আপনার সাম্প্রতিক সময়ের তামাক ছেড়ে দেয়ার চেষ্টাকে ভালভাবে বর্ণনা করে।

[ ] আমি ছেড়ে দেবার কোন পূর্বপরিকল্পনা করিনি, এমনিতে ছেড়ে দিয়েছি।

[ ] আমি ছেড়ে দেবার পরিকল্পনা করেছিলাম একইদিন, যেদিন কিছুক্ষণ পরে ছেড়ে দিয়েছি ।

[ ] আমি ছেড়ে দেবার পরিকল্পনা করেছিলাম ছেড়ে দেবার একদিন আগে।

[ ] আমি ছেড়ে দেবার পরিকল্পনা করেছিলাম ছেড়ে দেবার কিছুদিন আগে।

[ ] আমি ছেড়ে দেবার পরিকল্পনা করেছিলাম ছেড়ে দেবার কয়েক সপ্তাহ আগে ।

[ ] আমি ছেড়ে দেবার পরিকল্পনা করেছিলাম ছেড়ে দেবার কয়েক মাস পূর্বে ।

৭১. আপনি কি কখনো তামাক ব্যবহার ছেড়ে দেবার জন্য নিচের পদ্ধতিগুলোর কোনটা ব্যবহার করেছেন? (যেগুলো প্রযোজ্য সবগুলো চিহ্নিত করুন)

[ ] পরামর্শ, তামাক নিরাময় কেন্দ্র।

[ ] নিকোটিন প্রতিস্থাপন থেরাপি, যেমন- প্যাচ বা গাম।

[ ] অন্যান্য প্রেসক্রিপশন ওষুধ, যেমন- বুপরোপিয়োন।

[ ] সনাতন ওষুধসমূহ, যেমন- আয়ুর্বেদিক, হোমিওপ্যাথিক, ইউনানী।

[ ] ছেড়ে দেবার সহায়তাদানকারী টেলিডাক্তার এর মাধ্যমে।

[ ] আমি নিজে থেকে ছেড়ে দিয়েছি, কোন কিছু ব্যবহার করিনি।

[ ] আমি কখনোই তামাক ছেড়ে দেবার চেষ্টা করিনি।

[ ] অন্যান্য, বর্ণনা করুন ....

৭২. নিচের কোনটি আপনার সম্পূর্ণরূপে তামাক ছেড়ে দেবার ইচ্ছাকে বর্ণনা করে? আপনি কি বলবেন আপনি............?

[ ] কখনোই ছেড়ে দেবেন আশা করেন না।

[ ] ভবিষ্যতে ছেড়ে দিতে পারেন, তবে আগামী ৬ মাসের মধ্যে নয়।

[ ] আগামী ৬ মাসের মধ্যে ছেড়ে দেবেন।

[ ] আগামী ৩০ দিনের মধ্যে ছেড়ে দেবেন।

৭৩. বিগত ১২ মাসে, আপনি কতবার ডাক্তার বা স্বাস্থ্য সেবা প্রদানকারীর কাছে গিয়েছেন রুটিন চিকিৎসা গ্রহন বা অসুস্থতা বা আঘাতজনিত কারণে?

|__||__||__|বার

৭৪. আপনি বিগত ১২ মাসে ডাক্তার বা স্বাস্থ্যসেবা প্রদানকারীর কাছে যখন গিয়েছিলেন, তখন কি তারা জানতে চেয়েছিল আপনি ধোঁয়াহীন তামাক ব্যবহার করেন কিনা?

[ ] হ্যাঁ

[ ] না

৭৫.আপনি বিগত ১২ মাসে ডাক্তার বা স্বাস্থ্যসেবা প্রদানকারীর কাছে যখন গিয়েছিলেন, তখন কি তারা কখনো আপনাকে ধোঁয়াহীন তামাক ছাড়ার পরামর্শ দিয়েছিল?

[ ] হ্যাঁ

[ ] না

৭৬. আপনি বিগত ১২ মাসে ডাক্তার বা স্বাস্থ্যসেবা প্রদানকারীর কাছে গেলে তারা কি আপনাকে ধোঁয়াহীন তামাক ছাড়ার জন্য সহায়তা করেছিল, যেমন তামাক ছাড়ার পদ্ধতি সম্পর্কে কোন নির্দিষ্ট পরামর্শ বা ওষুধের প্রেসক্রিপশন?

[ ] হ্যাঁ

[ ] না

৭৭. আপনি বিগত ১২ মাসে ডাক্তার বা স্বাস্থ্যসেবা প্রদানকারীর কাছে যখন গিয়েছিলেন, তখন কি তারা কখনো তামাক ছাড়ার জন্য তাদের কাছ থেকে নিয়মিত চিকিৎসার ব্যবস্থা করেছিল বা আপনাকে কি তামাক নিরাময় কেন্দ্রে যেতে বলেছিল?

[ ] হ্যাঁ

[ ] না

৭৮. দয়া করে নিচের সেই সংখ্যাটি বাছাই করুন যা আপনার মতামতকে সবচেয়ে ভালভাবে বর্ণনা করবে। আপনি যদি তামাক ব্যবহার অব্যাহত রাখেন, তাতে আপনার মুখের ক্যান্সার হওয়ার সম্ভাবনা কতটুকু বলে আপনি মনে করেন?

সম্ভাবনা নেই	খুবই কম	কম	মাঝারি সম্ভাবনা	সম্ভাবনা আছে	খুবই সম্ভব	অবশ্যই হতে পারে
১	২	৩	৪	৫	৬	৭

৭৯. দয়াকরে নিচের সেই সংখ্যাটি বাছাই করুন যা আপনার মতামতকে সবচেয়ে ভালভাবে বর্ণনা করবে: আপনি যদি তামাক ব্যবহার অব্যাহত রাখেন, তাতে আপনার হৃৎপিন্ডের অসুখ হওয়ার সম্ভবনা কতটুকু বলে আপনি মনে করেন?

সম্ভাবনা নেই	খুবই কম	কম	মাঝারি সম্ভাবনা	সম্ভাবনা আছে	খুবই সম্ভব	অবশ্যই হতে পারে
১	২	৩	৪	৫	৬	৭

৮০. আপনার মতে, ধূমপানের চেয়ে ধোঁয়াহীন তামাক ব্যবহারে.......

[ ] স্বাস্থ্য ঝুঁকি বেশি

[ ] স্বাস্থ্য ঝুঁকি কম

[ ] একই রকমের স্বাস্থ্য ঝুঁকি

৮১. বিগত বছরে আপনি কতসময় আপনার ধোঁয়াহীন তামাক ব্যবহার কমাতে চেয়েছেন আপনার স্বাস্থ্য ঝুঁকি কমাবার জন্য?

[ ] কখনোই না

[ ] খুবই কম

[ ] মাঝে মাঝে

[ ] প্রায়ই

[ ] সবসময়

<u>আগের ধূমপানের ইতিহাস ঃ-</u>

আপনাকে এখন আপনার ধূমপানের ইতিহাস সম্পর্কে জানতে চাইব যার মধ্যে বিড়ি, সিগারেট, চুরুট, প্যাঁচানো সিগারেট, ভুট্টার পাতা বা নিউজপ্রিন্টের ভিতর প্যাঁচানো তামাক, হুঁকা, পাইপ, ছিলিম, চুট্টা ইত্যাদি। *দয়া করে <u>ধোঁয়াহীন তামাকের</u> জন্য এখন উত্তর দেবেন না।*

৮২. আপনি কি আপনার জীবনে কমপক্ষে ১০০টি সিগারেট গ্রহণ করেছেন?

[ ] হ্যাঁ

[ ] না

[যদি না হয়, এই অংশ বাদ দিয়ে যান]

৮৩. আপনি যখন প্রথম ধূমপান ব্যবহার করেছিলেন তখন আপনার বয়স কতছিল?

|__||__|বছর

৮৪. আপনি কি কখনো দৈনিক ৬ মাস বা তার চেয়ে বেশি সময় ধরে ধূমপান করেছেন?

[ ] হ্যাঁ

[ ] না

৮৫. আপনার জীবনের সেই সময়ের কথা স্মরণ করুন যখন আপনি সবচেয়ে বেশি ধূমপান করতেন। সেই সময়ে প্রতি সপ্তাহে আপনি কতদিন ধূমপান করতেন?

|__|দিন

৮৬.আপনার জীবনের সেই সময়ের কথা স্মরণ করুন যখন আপনি সবচেয়ে বেশি ধূমপান করতেন। সেই সময়ে যে দিন আপনি ধূমপান করতেন, দৈনিক কতবার/কতগুলো সিগারেট গ্রহণ করতেন?

|__||__|বার/সিগারেট প্রতিদিন

৮৭. আপনার জীবনের সেই সময়ের কথা স্মরণ করুন যখন আপনি সবচেয়ে বেশি ধূমপান করতেন। সেই সময় নিচের কোন পণ্যগুলো আপনি সাধারণত ব্যবহার করতেন? সবগুলো চিহ্নিত করুন যেগুলো প্রয়োজ্য।

[ ] প্যাকেটজাত সিগারেট

[ ] পাতা বা কাগজে হাতে প্যাঁচানো তামাক

[ ] বিড়ি

[ ] সিগার

[ ] সিগারিলো

[ ] চুরুট

[ ] পাইপ

[ ] হুকা

[ ] অন্য যে কোন কিছু, বর্ণনা করুন ....

৮৮. কতদিন যাবত আপনি ধূমপান ছেড়ে দিয়েছেন?

|__||__|মাস

|__||__|বছর

৮৯. আপনি কি নিচের কোন পদ্ধতি ব্যবহার করেছিলেন ধূমপান ছেড়ে দেবার জন্য? সবগুলো চিহ্নিত করুন যেগুলো প্রযোজ্য।

[ ] পরামর্শ, তামাক নিরাময় কেন্দ্র

[ ] নিকোটিন প্রতিস্থাপন থেরাপি, যেমন- প্যাচ বা গাম

[ ] অন্যান্য ওষুধের প্রেসক্রিপশন, যেমন- বুপরোপিয়ন

[ ] সনাতন ওষুধসমূহ, যেমন- আয়ুর্বেদিক, হোমিওপ্যাথিক, ইউনানী

[ ] ধুমপান ছাড়ার সহায়তাদানকারী টেলিডাক্তার এর মধ্যমে।

[ ] ধোঁয়াহীন তামাক ব্যবহার শুরু করে।

[ ] নিজে থেকে ছেড়ে দিয়েছি, কোন কিছু ব্যবহার করিনি।

[ ] অন্যান্য, বর্ণনা করুন...

৯০. আপনি কেন ধূমপান ছেড়ে দিলেন?

[ ] আমার অসুখ ধরা পড়েছিল।

[ ] ধূমপানের খরচ বেশি।

[ ] সবাই বারবার বলছে অথবা আমার ব্যাপারে মতামত দিচ্ছে ।

[ ] আমি ধূমপানে স্বাস্থ্যের উপর প্রভাবগুলো বুঝতে শুরু করেছিলাম।

[ ] আমার স্বাস্থ্য।

[ ] আমি দম বন্ধভাব দ্বারা ক্লান্ত হয়ে পড়ছিলাম।

[ ] আমি আমার জীবনের উপর নিয়ন্ত্রণ আনতে চাচ্ছিলাম।

[ ] আমি এর গন্ধ ও স্বাদে ক্লান্ত হয়ে পড়েছিলাম।

[ ] আমি জানি না, এমনিতেই করেছি।

[ ] বাসায় বা লোকসমাগমের মাঝে ধূমপান নিষিদ্ধ করা হয়েছে বা বাধা আরোপ হয়েছে।

[ ] অন্যান্য কারণসমূহ ....

স্বাস্থ্যের অবস্থা

আমি এখন আপনার স্বাস্থ্য সম্পর্কে কিছু প্রশ্ন করব। কখনও কি কোন ডাক্তার বা স্বাস্থ্য সেবা প্রদানকারী বলেছে আপনার নিচের কোনটি হয়েছে?

৯১. হৃৎপিন্ডের ক্রিয়া বন্ধ, যা মায়কারডিয়াল ইনফার্কশন নামে পরিচিত?

[ ] হ্যাঁ

[ ] না

[ ] জানি না

৯২. বুকে ব্যথা বা করোনারি হৃৎপিন্ডের সমস্যা?

[ ] হ্যাঁ

[ ]না

[ ] জানি না

৯৩. অতিরিক্ত দুশ্চিন্তা বা রক্তচাপ বেড়ে যাওয়া?

[ ] হ্যাঁ

[ ] না

[ ] জানি না

৯৪. ক্যান্সার

[ ] হ্যাঁ

[ ] না

[ ] জানি না

৯৫. হাপাঁনী ?

[ ] হ্যাঁ

[ ] না

[ ] জানি না

৯৬. দাঁতের বা মাড়ীর সমস্যা?

[ ] হ্যাঁ

[ ] না

[ ] জানি না

৯৭. বিগত মাসে আপনার নিয়মিত কাজ ছাড়া কতদিন আপনি কোন শারীরিক ব্যায়াম বা কাজে অংশগ্রহণ করেছেন?

|__||__|দিন প্রতি মাসে

[যদি >০ হয়] যখন আপনি একাজে অংশগ্রহণ করেছিলেন, তখন কত মিনিট বা ঘণ্টা আপনি একাজে নিয়োজিত থাকতেন?

|__||__|মিনিট

|__||__|ঘণ্টা

৯৮. বিগত ৭ দিনে গড়ে আপনি দৈনিক কতগুলো ফল খেয়েছিলেন?

|__||__|গুলো

৯৯. বিগত ৭ দিনে গড়ে আপনি দৈনিক কতবার সবজি খেয়েছেন?

|__||__|বার

১০০. সাধারণভাবে আপনি বলবেন আপনার স্বাস্থ্য ....?

[ ] চমৎকার

[ ] খুব ভাল

[ ] ভাল

[ ] মোটামুটি

[ ] খারাপ
